# Segmented molecular design of self-healing proteinaceous materials

**DOI:** 10.1038/srep13482

**Published:** 2015-09-01

**Authors:** Veikko Sariola, Abdon Pena-Francesch, Huihun Jung, Murat Çetinkaya, Carlos Pacheco, Metin Sitti, Melik C. Demirel

**Affiliations:** 1 Carnegie Mellon University, Department of Mechanical Engineering, Pittsburgh, PA, 15213, USA; 2 Aalto University, Department of Electrical Engineering and Automation, Espoo, 02150, Finland; 3 Pennsylvania State University, Department of Engineering Science and Mechanics, University Park, PA, 16802, USA; 4 BASF SE, Carl-Bosch Strasse 38, Ludwigshafen, 67056, Germany; 5 Pennsylvania State University, Department of Chemistry, University Park, PA, 16802, USA; 6 Max Planck Institute for Intelligent Systems, Physical Intelligence Department, Stuttgart, 70569, Germany; 7 Pennsylvania State University, Materials Research Institute and Huck Institutes of Life Sciences, University Park, PA, 16802, USA

## Abstract

Hierarchical assembly of self-healing adhesive proteins creates strong and robust structural and interfacial materials, but understanding of the molecular design and structure–property relationships of structural proteins remains unclear. Elucidating this relationship would allow rational design of next generation genetically engineered self-healing structural proteins. Here we report a general self-healing and -assembly strategy based on a multiphase recombinant protein based material. Segmented structure of the protein shows soft glycine- and tyrosine-rich segments with self-healing capability and hard beta-sheet segments. The soft segments are strongly plasticized by water, lowering the self-healing temperature close to body temperature. The hard segments self-assemble into nanoconfined domains to reinforce the material. The healing strength scales sublinearly with contact time, which associates with diffusion and wetting of autohesion. The finding suggests that recombinant structural proteins from heterologous expression have potential as strong and repairable engineering materials.

Self-healing materials are able to partially or completely heal damage inflicted on them, in particular by repairing cracks^1^,^2^,^3^,^4^,^5^,^6^,^7^,^8^,^9^. Stiff materials that self-heal in wet environment would greatly benefit biomedical applications, in particular by extending the lifetime of implants, but most self-healing polymeric chemistries are not suitable for aqueous environments^10^. Stiffness also comes at the cost of dynamic healing: weak intermolecular interactions result in a less stiff material, but more dynamic healing, and vice versa^3^,^4^. This tradeoff lead to the proposal of a general self-healing strategy based on a multiphase material: material should have hard and soft phases, with the self-healing capability embedded in the soft phase^3^,^4^. Although that strategy has been proposed earlier^3^,^4^, multiple step synthesis and polydisperse molecular weight distribution remain challenges for materials synthesis. Recombinant expression, however, provides industrial scale production without modifying the polymerization steps and with monodisperse molecular weight.

Underwater self-healing materials can be found in nature^11^, and significant research efforts have focused on mimicking the interfacial chemistry of marine barnacles^12^,^13^. Yet self-healing proteins are also known to self-assemble through supramolecular organization and the understanding of the role of this supramolecular self-assembly in the self-healing process remains limited. We propose that Nature uses this supramolecular self-assembly to achieve stiff self-healing structural proteins with soft/hard domain separation (Fig. 1A).

Recently, evidence of supramolecular self-assembly was found in the protein complex of squid ring teeth (SRT)^14^,^15^. SRT-based materials were shown to have several interesting properties as multifunctional engineering materials: a) high elastic modulus (6–8 GPa in air^14^, 2–4 GPa underwater^16^); b) capable of forming a strong adhesive bond under water^17^; and c) a reversible glass-to-rubber transition^18^. The rubbery properties are achieved in absence of any covalent cross-linking, but are a result of physical cross-linking between β-sheet segments, which self-assemble into nanoconfined domains^14^. In the ring teeth of European Common Squid (*Loligo vulgaris*), at least seven different proteins were identified, and primary sequences^18^ were obtained, which show a segmented copolymer architecture. The yield of this proteinaceous material from natural sources is low (~1 g of SRT from a 5 kg of squid) and the composition of the native material varies between squid species^19^. To have a sustainable material source and strict control over the protein composition, next generation sequencing (NGS) and heterologous recombinant protein expression were proposed^14^,^18^. Previously, we used differential scanning calorimetry (DSC) and dynamic mechanical analysis (DMA) to show that a recombinant 18 kDa SRT protein from *Loligo vulgaris* (LvSRT-18kDA) has a glass transition temperature of *T*_*g*_ ≈ 34 °C when plasticized by water. The material could be molded into various shapes in its rubbery phase or alternatively cast at room temperature by self-assembly in polar solvents^18^. Using DMA, the dry material was shown to have a stable storage modulus of *E’* ≈ 1 GPa and a loss modulus of *E”* ≈ 50 MPa until the degradation temperature of *T*_*d*_ ≈ 200 °C^18^. Those values are comparable to high density polyethylene (*E’* ≈ 1 GPa, *E”* ≈ 80 MPa at 38 °C^20^).

In this paper, we report self-healing of this recombinant protein, which is achieved in mild conditions, by pressing in rubbery state (shear thinning) in aquatic media. Spherical probe adhesion tests show sublinear time dependence (≈t^1/2^) and a critical glass transition temperature (*T*_g_) above which crack-healing in the material can be repeated multiple times. We elucidate the segmented copolymer structure of the protein, by showing that the soft amorphous regions play an important role in the self-healing of the material, while the hard nanoconfined β-sheet domains reinforce the structure.

## Results and Discussion

**Figure 1:**
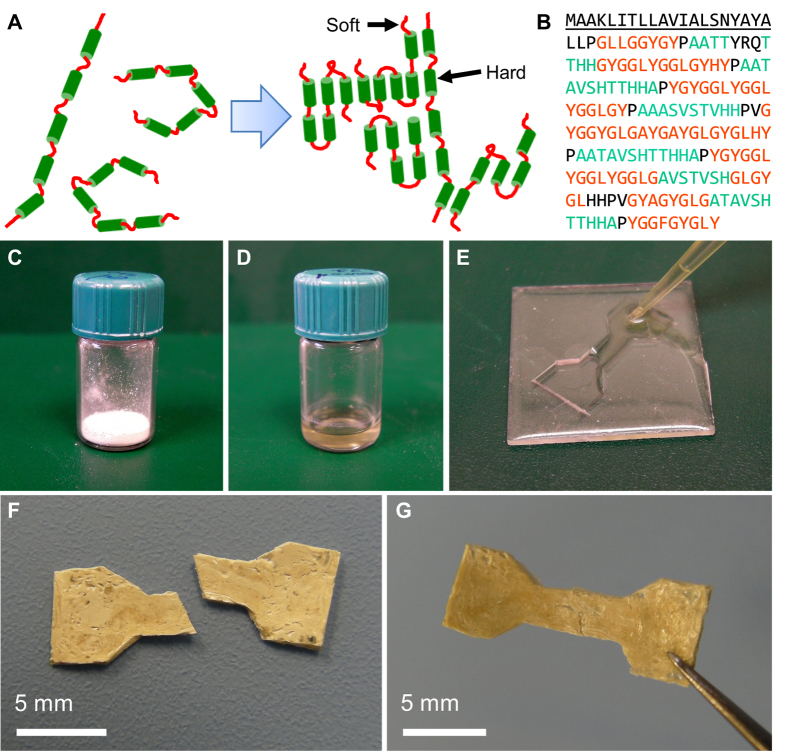
Sequencing and heterologous expression of the self-healing thermoplastic squid ring teeth protein. (**A**) Schematic of supramolecular self-assembly. (**B**) The amino acid sequence of the protein showing a segmented copolymer structure. The single letter amino acid abbreviations follow the standard convention. Self-assembling β-sheet regions are colored green and amorphous regions red. (**C**) The 18 kDa recombinant protein powder is obtained using next generation sequencing and heterologous expression. (**D**,**E**) The recombinant protein dissolves in polar solvents (**D**) and can be cast into various shapes (**E**). The material self-assembles upon solvent evaporation. (**F**) Dog bone shaped recombinant protein beam after cutting in half. (**G**) The beam after pressing together in warm 45 °C water.

The amino acid sequence, from NGS^18^, is shown in Fig. 1B. The segmented copolymer blocks are often divided by proline residues. The glycine-rich regions are amorphous, while the alanine, valine, serine, threonine, and histidine (AVSHT) rich regions are capable of forming β-sheets^18^. The recombinant expression is performed by cloning the LvSRT gene into pET14b vector (Novagen) and transformed into *E. coli* as described earlier^18^. Single step purification of LvSRT-18kDA protein yields of ~0.5 g/L in biomass (Fig. 1C). The size of the resulting protein was confirmed using SDS-PAGE (Fig. S1). SDS-PAGE is typically sensitive to proteins with distinct molecular weight and with concentration more than 10%. Since no side bands were observed, the purity of the protein is estimated to be at least 90%; however, molecules with similar molecular weights cannot be distinguished using the method. The protein can be dissolved in polar protic solvents (Fig. 1D), especially 1,1,1,3,3,3-hexafluoro-2-propanol (HFIP). The material can be cast into various shapes by self-assembly upon solvent evaporation (Fig. 1E). Up to 100 mg/ml could be dissolved in HFIP until the viscosity of the solution becomes unsuitable for casting.

**Figure 2:**
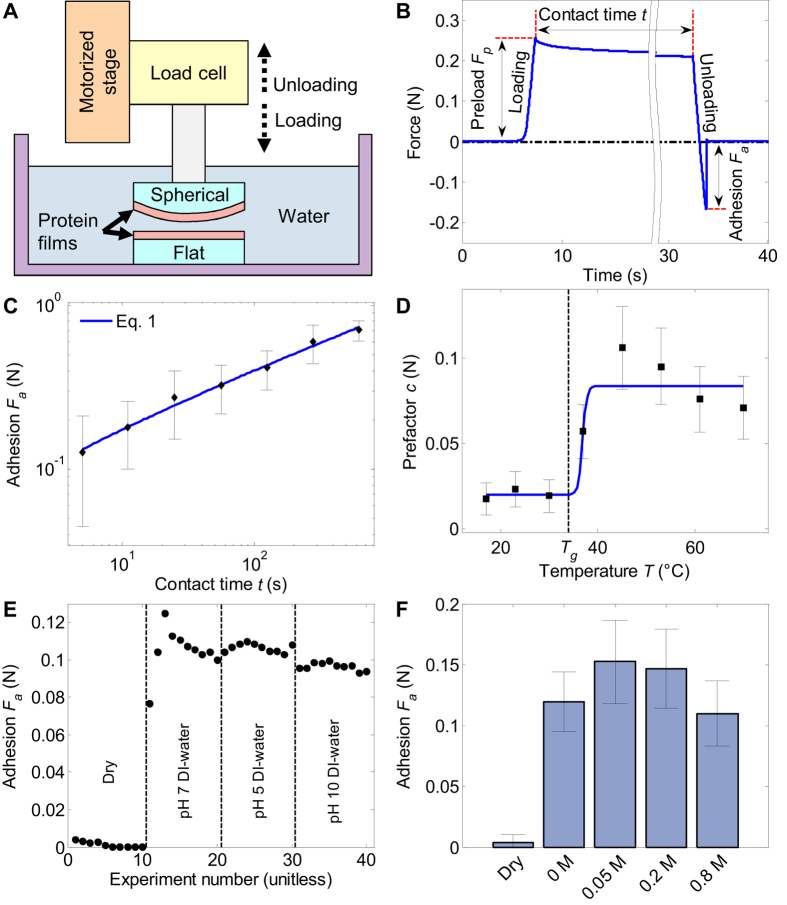
Self-healing of recombinant 18 kDa squid ring teeth protein. (**A**) Schematic of the spherical probe adhesion tests used to characterize the self-healing between protein films. A spherical indenter was loaded and unloaded against a flat surface submerged in deionized water. Both surfaces were coated with 10 μm thick protein film. The radius of curvature of the spherical surface was 20.67 mm. (**B**) An example of the measured force signal from an adhesive experiment. The preload force *F*_*p*_ was 0.25 N in all experiments. (**C**) Plot of *F*_*a*_as a function of *t* at *T* = 45 °C. The data is captured by a power law regression model (Eq. 1). Error bars show the standard deviation of the measurements (repetitions: 6). (**D**) Prefactor *c(T)* as a function of temperature. Error bars show the 95% confidence interval from the parameter estimation. (**E)** Repeatability and effect of pH on the autohesion at 70 °C. (**F)** Autohesion in varying NaCl concentrations. Error bars show the standard deviation of the measurements (samples: 2, repetitions per sample: 10).

Qualitatively, autohesive self-healing was observed in a fractured part. After casting, a dog bone sample was cut in two pieces (Fig. 1F). When placed in warm 45 °C water, the material softened and pressed back together (Fig. 1G). Quantitative characterization of the self-healing was done by measuring the forces and energies needed to separate two protein surfaces after they have been joined together. To this end, we used spherical probe adhesion tests^21^, where a protein-coated glass sphere (radius of curvature *R* = 20.67 mm) was pressed against a protein-coated flat glass surface under water (Figs 2A and S2). The film thickness was approximately 10 μm. The surfaces were pressed together with a preload force of *F*_*p*_ = 0.25 N for a contact time of *t*, and the adhesion force, *F*_*a*_, was recorded during retraction (Fig. 2B). By varying the temperature, *T*, from 17 °C (<*T*_*g*_) to 70 °C (>*T*_*g*_) and time, *t*, logarithmically between 5 s and 625 s, we find that *F*_*a*_ drops significantly when *T* < *T*_*g*_ (under water). Of all the statistical regression models considered (Table S1), we found that the data (Fig. S3) was best captured by a power law of


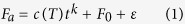


where *c* is a temperature dependent prefactor, *t* is in seconds, *k* = 0.36 ± 0.02, and *F*_*0*_ = −0.04 ± 0.03 N are temperature-independent constants, and *ε* is the residual (Fig. 2C). At temperatures 17–30 °C < *T*_*g*_, we measured very little adhesion; consequently, *c* ≈ 0 N. Around 37 °C ≈ *T*_*g*_, *c* starts to increase significantly. Notice that the negative *F*_*0*_ is unphysical and may be an artifact of the regression modeling or a systematic measurement error; the second-best regression model considered was 
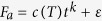
.

An ideal elastic model relates *F*_*a*_ to the critical strain energy release rate *G*_*c*_ through the Johnson-Kendall-Roberts (JKR) theory with *F*_*a*_ ~ *G*_*c*_ for a spherical probe and *F*_*a*_ ~ *G*_*c*_^1/2^ for a cylindrical flat probe^21^,^22^. Our protein film is viscoelastic, so these relations are not expected to hold. Nevertheless, assuming that the relationship is in between of a spherical and a cylindrical probe, for large *t* we find *G*_*c*_ ~ *t*^0.34–0.76^, a range that includes the often found *G*_*c*_ ~ *t*^1/2^, a power-law associated with the diffusion and wetting models of autohesion (self-bonding)^23^. For macromolecules, it is important to distinguish between macro-Brownian motion (diffusion of molecules) and micro-Brownian motion (diffusion of segments)^24^. The existence of crosslinks in the material (intermolecular β-sheet links in our case) could inhibit macro-Brownian motion^23^, leaving solid/solid wetting and micro-Brownian as the plausible autohesion mechanisms for the recombinant LvSRT protein.

To investigate the importance of media for the self-healing, we repeatedly measured autohesion with *t* = 25 s and *T* = 70 °C in different media such as dry, deionized water (DI) water with varying pH (5, 7 and 10), and DI water with varying concentrations of NaCl between 0.0 and 0.8 M. The high temperature of *T* = 70 °C was chosen to ensure full transition to the rubbery state. The results from pH experiments are summarized in Fig. 2E. There is a dramatic increase in autohesion after adding water, which is expected due to lowered *T*_*g*_ by plasticizing effect of the water. The first few experiments in water shows a large dispersion in the measurements, indicating plastic deformations in the films when repeatedly probing the same point, but in general, the adhesion was not sensitive to mild changes in the pH. In alkaline conditions (pH 10), the adhesion drops slightly, which is consistent with the previously observed drop in adhesion of the native SRT protein complex on glass when pH > 10. The observed drop in adhesion is consistent with β-sheet nanodomains becoming disrupted in alkaline conditions. The effect of added NaCl on the autohesion is summarized in Fig. 2F. No significant change in autohesion is seen when the ion concentration is increased. We previously reported that the steady state adhesion (*t* ≈ days) of native SRT to glass slides is reduced when the ion concentration is increased; this does not contradict our current results, but rather shows that the steady state adhesion strength of SRT to glass is limited by hydrogen bonding, while other autohesive phenomena (e.g., chain entanglements or wetting) limit the dynamic adhesion strength.

To understand effect of diffusion as well as intermolecular interactions in the amorphous domain as a function of temperature, we performed molecular dynamics simulations. We obtained force-extension curves for the amorphous region of the LvSRT-18kDA protein (Fig. S4A). We here chose the repeated motif YGYGGLYGGLYGGLGY. This particular sequence is chosen because it represents a composition that is high in glycine (flexibility) and tyrosine (hydrogen bonding^25^) accounting for more than 60% of total amino acids. The hydrogen bond networks formed between peptide chains throughout a simulation is measured by autocorrelation function (ACF) shown in Fig. S4B. The figure clearly shows a drop at 320 K, which agrees with the observed glass transition temperature in DSC experiments. Hence, it is clear that both hydrogen bonding and chain entanglement in amorphous domains are playing a role in the adhesion mechanism.

**Figure 3:**
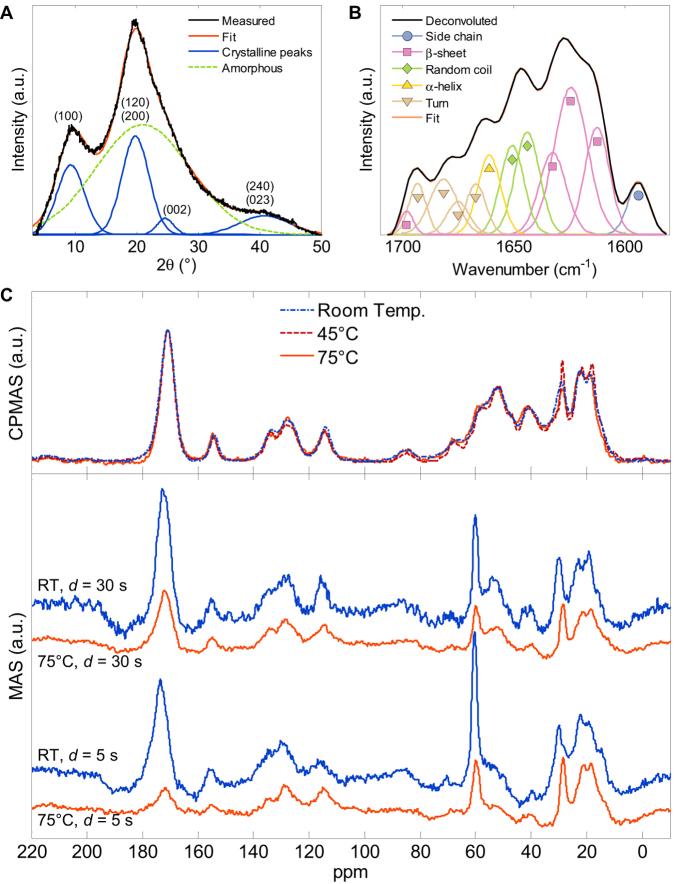
Characterization of the nanoconfined β-sheet domains. (**A**) XRD spectrum with peak intensities labeled with crystallographic directions. (**B**) FTIR spectrum of the amide band. (**C**) Comparison of ^13^C-NMR spectra at room temperature (RT), 45 °C and 75 °C and two different relaxation delays *d* in wet conditions.

We then elucidate the role of the self-assembled β-sheet domains in the self-healing material using X-ray diffraction (XRD). Even after the film is heated to rubbery state in water and then dried, XRD confirms the existence of ≈3 nm ordered domains (Fig. 3A), according to the Scherrer equation^26^. Similar results have been previously obtained for the Humboldt squid proteins^15^. To confirm the nature of these nanodomains, we also used Fourier transform infrared spectroscopy (FTIR) and found a large amide I band at around 1640 cm^−1^ (Figs 3B and S5). Analysis of the Fourier self-deconvoluted spectrum of the amide I band^27^,^28^ shows that that most contributions to this band are primarily from β-sheets and secondarily from random coil and turn conformations. Similarly to silk^28^, a peak around 1625 cm^−1^ is assigned to stacked antiparallel β-sheet structures. In Fig. 3B, the beta sheet bands in the 1610–1630 cm−1 region, consistent with the antiparallel arrangement. Furthermore, the considerable amount of turns also favors the antiparallel arrangement.

In a semicrystalline polymer, co-crystallization can give rise to a very large increase in the autohesive adhesion energy^29^. In co-crystallization, chains of the same nature, that are initially on opposite sides of the interface, crystallize in a single crystallite. To inquire the effect of temperature on the β-sheet nanodomains in wet environments, we used solid-state carbon-13 nuclear magnetic resonance (^13^C-NMR). Cross-polarization magic angle spinning (CP-MAS) shows almost no change when the wet material is heated from room temperature to 75 °C (Fig. 3C), and the spectrum is very similar even when the material is dry (Fig. S6). CP-MAS is sensitive only to ordered (i.e., β-sheet) domains of the material, showing that there is little change in the ordered domains upon heating. On the contrary, ordinary magic angle spinning (MAS) ^13^C-NMR in sensitive to both ordered and unordered (i.e., amorphous regions of the material), and shows shift and intensity changes in the spectra upon heating the wet material (Fig. 3C). Further comparing MAS spectra with short (5 s) and long (30 s) relaxation delays *d*, we find that the carbonyl peak around 172 ppm with short relaxation delay is relatively shallower at 75 °C compared to room temperature, but the difference is less pronounced with the long relaxation delay. β-sheet domains should have longer *T*_*1*_ relaxation times, so most change in the carbonyl peak can be attributed to the amorphous regions with mostly glycine residues. Overall, both MAS and CPMAS results are consistent with the assumption that most changes upon heating are in the amorphous regions of the material, while the ordered domains remain intact at 75 °C. Consequently, we find it unlikely that co-crystallization contributes to the self-healing.

Finally, we compared the self-healing behavior of the 18 kDa protein to a recombinant 22 kDa protein with similar two-segmented architecture and mechanical properties (E’ ≈ 1 GPa in dry). The main difference between the proteins is that the segment borders and repeat motifs are less clear in the 22 kDa protein (Fig. S7A). Nevertheless, the 22 kDa protein shows comparable time-behavior in the adhesion measurements (Fig. S7B). This confirms that the general hard/soft design is more important for self-healing than individual motifs or their locations.

In summary, self-healing high strength structural proteins may provide novel mechanical properties for clinical applications such as orthopedic devices for repair^30^, and treatment strategies as novel biodegradable matrices to deliver and stimulate bioactive molecules for wound healing.

## Methods

### Recombinant protein expression

A single colony was inoculated and grown overnight in 250 ml of LB with ampicillin (100 μg/ml). The overnight culture was inoculated into an 80 L fermenter containing LB/ampicillin (100 μg/ml). The culture was grown at 37 °C to an OD600 of 0.6 when IPTG was added. The cells were then pelleted at 10,000 rpm. for 15 min and washed twice with 20 mM Tris buffer pH 8. The cell pellet was then resuspended in 50 ml of lysis buffer (50 mM Tris pH 7.4, 200 mM NaCl, 1 mM PMSF, 10 mg/ml lyzosyme, 0.1 mg/ml DNAse I, and 2 mM EDTA) and lysed using a high-pressure homogenizer (Microfluidics M-110EH-30 microfluidizer). The lysed cells were pelleted at 14,000 rpm. for 1 h at 4 °C. The pellet was then washed twice with urea extraction buffer (100 mM Tris pH7.4, 5 mM EDTA, 2 M urea, 2% (v/v) Triton X-100) and then twice with a wash buffer (100 mM Tris pH7.4, 5 mM EDTA). After washing step, recombinant proteins dried using a Freezezone 12 (Labcono, Kansas city, MO). Recombinant proteins of ~90% purity were obtained in this manner and yields were estimated at ~0.5 g/L.

### Healing dog bone shaped sample

20 mg of recombinant 18 kDa SRT protein (Fig. 1C) was dissolved in 1,1,1,3,3,3-hexafluoro-2-propanol (HFIP, Fig. 1D) and cast on a flat surface. After solvent evaporation in chemical fume hood, the SRT films were rinsed with DI water. The protein was molded into a 15 mm dog bone shape in a PDMS mold (Fig. 1E) at 75 °C with DI water in excess under 1 MPa of pressure for 10 minutes. After molding, the sample was dried in ambient conditions and was cut in half with a razor blade (Fig. 1F). The two parts were pressed back together in the mold (at 45 °C > T_g_) by adding 10 mL of DI water to the cut area and applying 1 MPa for 10 minutes (Fig. 1G).

### Solvent casting films

For the adhesion measurements, flat 1 mm thick microscope glass slides and spherical plano-convex lenses (Edmund-Optics #45–095, uncoated, *R* = 20.67 mm) were coated by solvent cast protein films (Fig. S1, inset). The slides were first cut into approximately 20 mm × 25 mm pieces and wiped clean using isopropyl alcohol. The lenses were reused in several experiments, and between the experiments, the lenses were thoroughly cleaned using ultrasound energized acetone, HFIP and isopropyl alcohol. The purified recombinant protein was dissolved in HFIP (20 mg/ml). 40 μl of solution was dispensed on both substrates, and the solvent was left to evaporate on a 65 °C hot plate for at least 10 minutes in a chemical fume hood. Then, another 40 μl was dispensed on both substrates and the samples were left to dry on the hotplate for at least 30 minutes. The final mean thickness of the film was estimated to be 10 μm; however, distinct ring stain pattern^31^ could be observed and the films were always probed near the center, so the effective film thickness under the contact area was much smaller.

### Adhesion measurements

A protein-coated flat glass slide was mounted using a cyanoacrylate adhesive (Loctite 498) to the bottom of a 35 mm petri dish and the spherical glass lens was mounted to an aluminum adapter. The aluminum adapter was threaded and screwed onto a load cell (Transducer Techniques GSO-250). The sensor noise level of the load cell was less than 1 mN. The petri dish was filled with 4 ml of deionized water. A K-type thermocouple (accuracy ≈ 1 °C) was immersed in the water and based on the measured temperature, a proportional-integral-differential controller regulated the temperature of the water using external heating and cooling elements. A computer-controlled motorized stage (Newport MFA-CC) was used to load and unload the spherical film against the flat slide, at a speed of 20 μm/s. Fig. S2 shows a photograph of the measurement setup. A computer recorded the load cell signal using a signal acquisition card and was responsible for controlling the motorized stage. Each experiment consisted of loading until preload was reached, waiting for contact time, *t*, and then unloading. Preload was kept constant (0.25 N) in all tests. Effects of buoyancy (<1 mN) and capillary forces of the air-liquid interface (<2 mN) were visible in the load cell signal, but their effect on the measurements was minor. Adhesion was measured as the maximum tensile force measured during unloading. When repeating the measurement with one sample, the measurement reproducibility is within 2 mN. The adhesion was measured at eight different temperatures: 17, 23, 30, 37, 45, 53, 61, and 70 °C. One sample combination (lens and flat slide) was used for each temperature. At each temperature, seven different contact times were tested: 5 s, 11 s, 25 s, 56 s, 125 s, 280 s, and 625 s. Each contact time was tested six times and the experiments were ordered in a balanced Latin square order (Table S2), to minimize carry-over effects from probing the same spot repeatedly. The raw data from these measurements is plotted in Fig. S3. For most temperatures, there was a slight increase in adhesion with repeated experiments; this was likely due to increase in contact area due to plastic deformations in the film. The flat films were observed after the experiments and from some of them, the probing point could be located, further indicating that plastic deformations occurred during the probing. For the pH experiments, one sample pair was probed (T = 70 °C, t = 25 s) in dry, DI-water, DI-water with pH 5 and DI-water with pH 10, in that order. Between the experiments, the media was changed and let to equilibrate back to 70 °C, which took approximately 15 minutes. The measurement was repeated 10 times in each media. HCl and NaOH were used for titration. Similarly, varying NaCl concentrations were tested in different media: dry, DI water, DI water with 0.05 M NaCl, DI water with 0.2 M NaCl, and DI water with 0.8 M NaCl, respectively. The NaCl experiments were repeated with four samples.

### Molecular dynamics simulations

All molecular dynamics (MD) simulations (Fig. S4) were performed with Gromacs simulation engine v.4.5.5^32^ using OPLS-AA force field^33^ and the SPC water model^34^. Periodic boundary conditions were applied in all directions along with Particle-Mesh Ewald summations^35^ for long-range electrostatics (>1 nm). Van der Waals forces were modified with a shift potential. The integration time step was 2 fs. V-rescale type of thermostats for peptide and water molecules and a single Parrinello-Rahman type of barostat (isotropic, 1 bar) were used for temperature and pressure couplings, respectively. Bond constraints were applied with the LINCS algorithm. The simulated amorphous peptide sequence contained 16 amino acids: YGYGGLYGGLYGGLGY. A single peptide chain was first built as a coil with neutral N- and C-termini. The chain was then energy minimized in vacuo using the steepest-descent algorithm. Five more copies of this peptide chain were added into the simulation box and then arranged in a randomly coiled/entangled state in order to mimic a polymer-melt type of morphology. The peptide chains were once more energy minimized and then simulated in vacuo with MD for 4 ns for a fast removal of the most unwanted geometric conformations. These chains were then transferred to a rectangular box with 20 nm × 8 nm × 8 nm size and solvated with 42,317 SPC type of water molecules at pH = 7. No salt ions were added in order to mimic the experimental conditions. After another round of energy minimization, the solvated box was equilibrated for 10 ns using an NPT ensemble at 1 bar pressure and 300 K temperature. After equilibration, the 6 solvated peptide chains were pulled away from each other along x-axis using steered MD with an NVT ensemble at a temperature range covering 280 K to 330 K with 10 K intervals. The peptide chains were pulled away starting from their coiled conformation in such a way that 3 chains were pulled in –x direction, while the other 3 in + x direction with a speed of 1 nm/ns. In total, 2 harmonic springs with 500 kJ/mol/nm^2^ stiffness were placed at the center of mass of 3 backbone termini atoms belonging to the 3 peptides pulled in a particular direction. The displacement of the harmonic springs with respect to their reference positions and the resulting pull forces were recorded during the simulation. The integrals of these force-displacement curves were calculated for estimating the toughness, i.e. the required energy input to separate the peptide chains from each other. Moreover, the number of peptide-peptide hydrogen bonds (with 0.35 nm distance and 30 degree angle thresholds) and their autocorrelation functions (ACF) were calculated using the trajectory output. According to the definition by van der Spoel *et al.*^36^, the ACF assumed that the hydrogen bonds were of interrupted type, i.e., they were allowed to break and re-form during the simulation. An ACF curve indicated the relevance between the hydrogen bond networks formed between peptide chains throughout a simulation. In other words, they showed how stable the peptide-peptide hydrogen bond networks were at a certain temperature.

### X-ray diffraction spectroscopy

X-ray diffraction (WAXS) data was collected from 500 μm SRT films in a Rigaku DMAX-Rapid II Microdiffractometer using Cu Kα source and 30 μm collimator with 10 minute exposure at 50 kV and 40 mA. The data is analyzed with MDI Jade X-ray diffraction software.

### Attenuated total reflectance Fourier transform infrared spectroscopy

Spectral data (Fig. S5) was collected (Thermo Scientific Nicolet 6700 FT-IR) under attenuated total reflection (diamond crystal) mode using Happ-Genzel apodization with 4 cm^−1^ resolution from wavenumber 400 to 4000 cm^−1^. For each spectrum, 256 scans are co-added.

Fourier self-deconvolution (FSD) and second derivative of the amide I band (1580–1706 cm^−1^) was performed by OMNIC software (Thermo Scientific, v7.3). Second derivative was obtained from the original amide I spectra and a nine-point Savitsky-Golay smoothing filter of polynomial degree 5 was applied. FSD was performed with Lorentzian line shape with 25 cm^−1^ bandwidth and an enhancement factor of 2. Curve fitting was performed as described elsewhere^27^,^28^. Individual bands were fitted to the deconvoluted spectra^37^ and were assigned to secondary structural components^27^. The number and position of the fitted bands were obtained from the second derivative spectra, where the minima in the second derivative spectra corresponded to the fitted band maxima in the deconvoluted spectra. Curve fitting was performed in OriginPro 8.5 software by fitting of Gaussian by a nonlinear least-squares method. First, the initial band positions (taken from the second derivative) were fixed and the width and height were left as free parameter. Then the band positions were allowed to change within ±2 cm^−1^ range using the built-in Levenberg-Marquardt algorithm. The relative areas of the single bands were used in the calculation of the fraction of the secondary structure features.

### Nuclear magnetic resonance spectroscopy

Solid-state NMR measurements were done using Bruker Avance-300 with 1 H operational frequency of 300.43 MHz and 75.54 MHz for 13C running TopSpin 1.3. The instrument was equipped with a 4-mm H/X CPMAS probe. The sample was prepared in a zirconia rotor with Vespel Kel-F and spun at 6.5 kHz. The data acquisition parameters for the 13C MAS NMR spectra were: Bruker’s pulse program “hpdec.av”, 2k points with acquisition time of 20 ms and spectral width of 50 kHz, 13C pulse of 4 μs, relaxation delay of 5 and 30 sec, and 8,500–11,500 scans. The 13C data were acquired with a 1 H decoupling with 66 kHz SPINAL-128. For the 13C CPMAS NMR spectra, Bruker’s pulse program was “cp.av”, delay of 5 sec, and contact time of 1 ms. The other parameters were same as the MAS spectra. The NMR data were processed with LB = 30 Hz and zero-filled to 32k points. When testing the material in wet conditions, it was fully saturated with water.

## Additional Information

**How to cite this article**: Sariola, V. *et al.* Segmented molecular design of self-healing proteinaceous materials. *Sci. Rep.* **5**, 13482; doi: 10.1038/srep13482 (2015).

## Supplementary Material

Supplementary Information
