# Supplementary material for: Segmented molecular design of self-healing proteinaceous materials
Source: Sci Rep. 2015 Sep 1;5:13482. doi: 10.1038/srep13482 (PMC4555047; doi:10.1038/srep13482)
Supplement: Supplementary Information [file srep13482-s1.pdf]

SUPPLEMENTARY MATERIALS

Title: Segmented molecular design of self-healing proteinaceous materials

Authors: Veikko Sariola[1,2]†, Abdon Pena-Francesch[3]†, Huihun Jung[3], Murat Çetinkaya[4], Carlos Pacheco[5], Metin Sitti[1,6], Melik C. Demirel[3,7]

Affiliations:

[1] Department of Mechanical Engineering, Carnegie Mellon University, Pittsburgh, PA, 15213, USA.

[2] Department of Electrical Engineering and Automation, Aalto University, Espoo, 02150, Finland.

[3] Department of Engineering Science and Mechanics, Pennsylvania State University, University Park, PA, 16802, USA.

[4] BASF SE, Carl-Bosch Strasse 38, Ludwigshafen, 67056, Germany.

[5] Department of Chemistry, Pennsylvania State University, University Park, PA, 16802, USA.

[6] Max Planck Institute for Intelligent Systems, Physical Intelligence Department, Stuttgart, 70659, Germany.

[7] Materials Research Institute and Huck Institutes of Life Sciences, Pennsylvania State University, University Park, PA, 16802, USA.

†Equally contributing authors.

Figures S1-S5

Table S1-S2

References

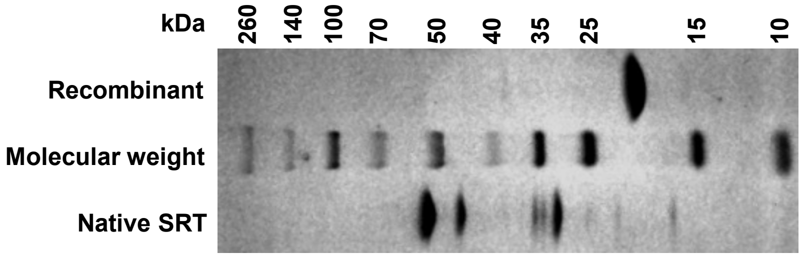

Fig. S1. SDS-PAGE (sodium dodecyl sulfate polyacrylamide gel electrophoresis) of the native and recombinant materials along with molecular weight markers.

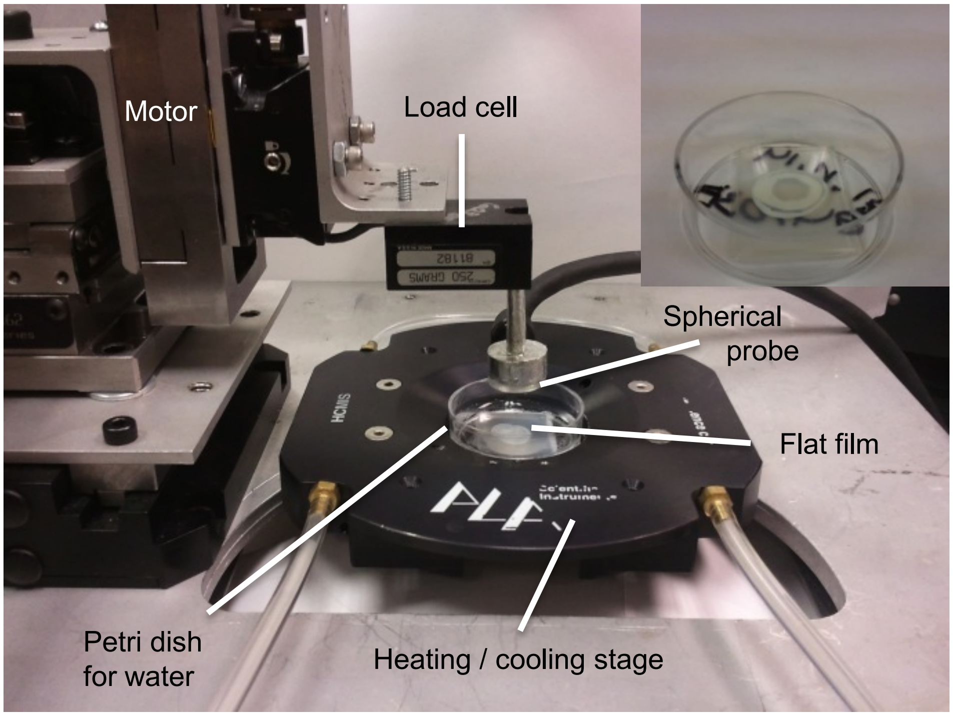

Fig. S2. Measurement setup for the adhesion experiments. The inset shows a close up of the flat protein film, where a clear ring stain pattern is visible.

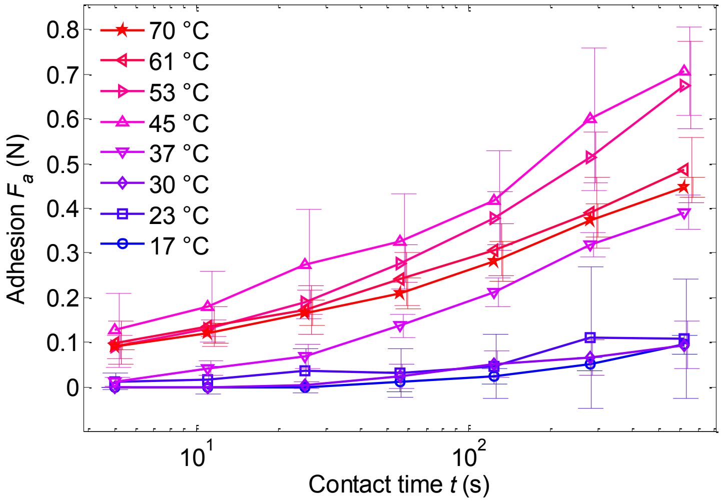

Fig. S3. Raw data used for the regression model (1). Error bars show standard deviation of measurements (n = 6).

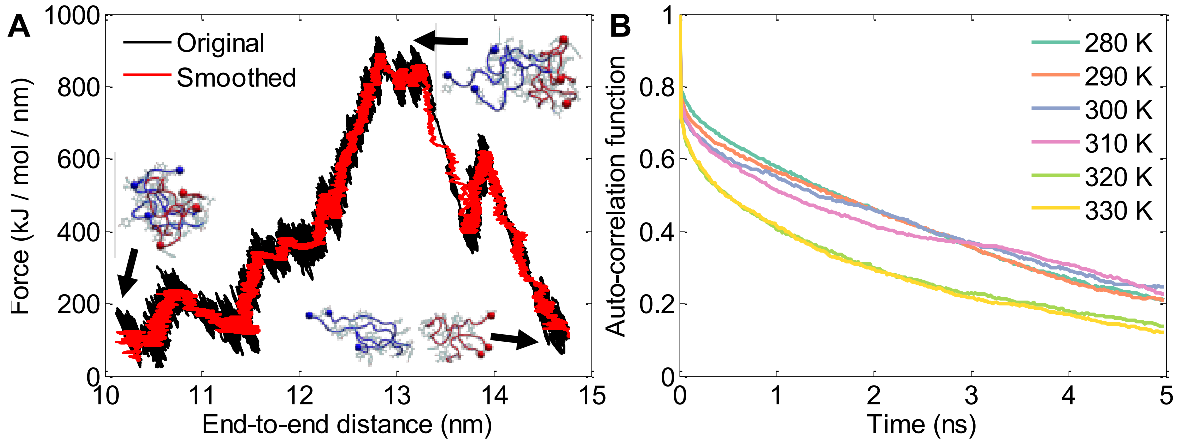

Fig. S4. Molecular dynamics simulations of chain pull-out. (**A**) A sample force-displacement curve from a steered MD simulation. The x-axis shows the distance between the two harmonic springs moving away from each other, while the y-axis shows the corresponding force imposed on the springs. The black data points represent the instantaneous force-displacement values and the red line shows the smoothed data. The inset figures are the snapshots of the peptide chains at the beginning, point of peak force and at the end of the simulation. It is worth mentioning that this curve belongs to a simulation performed in vacuo for the sake of demonstration. Simulations with solvated chains contain a higher noise to signal ratio and the pull forces are lower due to peptide-peptide hydrogen bonding replaced by the ones with water molecules. **(B)** The ACF profiles of peptide-peptide hydrogen bonding in solvated systems at different temperatures. The

sudden drop of ACF at 320 and 330K indicates the threshold of thermal fluctuations overcoming the hydrogen bond stability between peptides.

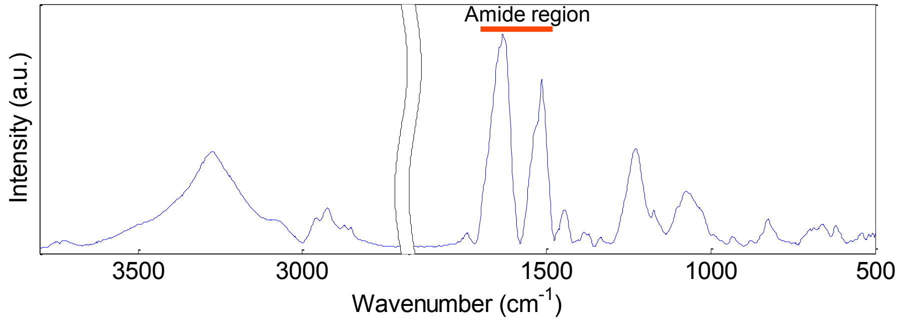

Fig. S5. Full FTIR spectrum of the recombinant protein. The amide I band of this spectrum was used for the peak fitting in Fig. 3B.

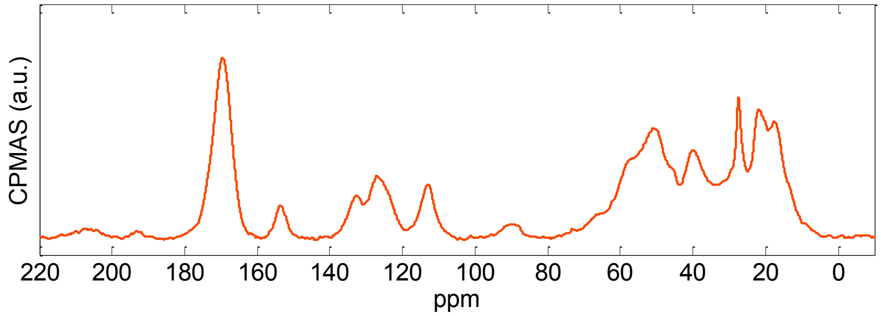

Fig. S6. ^{13}C-ssNMR CPMAS spectrum of the dry material at 70 ˚C.

A

MVATVLVIMSMIAALSCQ
SEAALSVGTSVKTIRQSV
HHGAVPAVGHTTVTHAVP
HAYAYGGLPYGDAFGGLY
GGLYGGLYGSPAATSVKT
VSHGFHPTLPVGSTISHT
THGVHHPVTYGGLGLGGL
GYGGLGYGGLGYGGLGYG
GLGYGGLGYGGLGYGGLG
AGGLYGLHYPGAVGLGYG
LGGGYGGLYGLHLPAATS
VSHTTHGVHHPALGLGLY
GGAHLPAASSVTHTTHGV
AHPGLGLNYGVYGLH

B

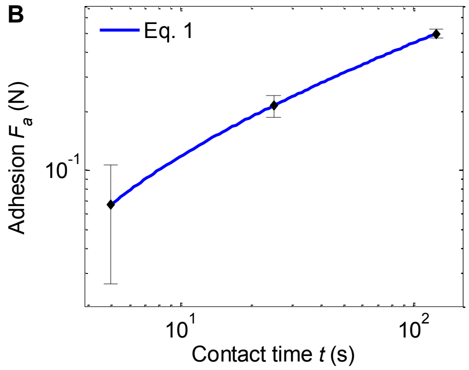

Fig. S7. Self-healing of recombinant 22 kDa squid ring teeth protein. (**A**) Full amino acid sequence of the recombinant 22 kDa squid ring teeth protein. The coloring shows similar blocks as the 18 kDa protein (Fig. 1B) but the segments are not as clearly separated as in the 18 kDa protein. (**B**) Plot of F_a as a function of t at T = 41 ˚C. Error bars show the standard deviation of the measurements (repetitions: 5). The contact time dependence was comparable to the 18 kDa protein (Fig. 2C). Best fit parameters for eq. 1 were k = 0.41, c = 0.08 N and F_0 = -0.09 N.

Table S1. Different regression models considered for the adhesion data. The response variable was kept always F_{ad}, to allow comparing different nonlinear regression models using Akaike Information Criterion (AIC), small-sample-size corrected version of AIC (AICc) and Bayesian Information Criterion (BIC). In general, AIC and AICc favor regression accuracy over model simplicity compared to BIC. The information criteria were computed as: $AIC = n \ln(RSS / n) + 2m$, $AICc = AIC + 2m(m + 1)/(n - m - 1)$ and $BIC = n \ln(RSS/n) + m \ln n$, where n = 336 is the total number of datapoints, RSS is the sum of square residuals and m is the number of parameters in the model. In the models, argument T denotes that the coefficient is temperature dependent, resulting in 8 parameters in the model instead of one (8 different temperatures were tested). The relative weights w are computed by giving each model i an absolute weight of $e^{-(x_i - \min x)/2}$, where x are the information criterion values. Based on BIC, $F_a = a(T)\, t^k + F_0 + \varepsilon$ was chosen as the best fit to the data. The regression analysis was done in Matlab, using function `nlinfit`, and the parameter 95%-confidence intervals in Fig. 2D were calculated using Matlab function `nlpredci`. When `nlinfit` failed to converge, the model was possibly over parameterized and the model was taken to have an absolute weight of 0. The coloring reflects the relative weights of the models (red: 0 – 0.0001, orange: 0.0001 – 0.01, yellow: 0.01 – 0.05, and green: 0.05 – 1).

Regression model	m	w_{AIC}	w_{AICc}	w_{BIC}
$F_a = ct^k + \varepsilon$	2	< 0.0001	< 0.0001	< 0.0001
$F_a = c(T)t^k + \varepsilon$	9	< 0.0001	0.00010	0.049
$F_a = ct^{k(T)} + \varepsilon$	9	< 0.0001	< 0.0001	< 0.0001
$F_a = c(T)t^{k(T)} + \varepsilon$	16	0.018	0.02	< 0.0001
$F_a = ct^k + F_0 + \varepsilon$	3	< 0.0001	< 0.0001	< 0.0001
$F_a = c(T)t^k + F_0 + \varepsilon$	10	0.0067	0.012	0.95
$F_a = ct^{k(T)} + F_0 + \varepsilon$	10	< 0.0001	< 0.0001	0.0012
$F_a = c(T)t^{k(T)} + F_0 + \varepsilon$	17	0.31	0.30	< 0.0001
$F_a = ct^k + F_0(T) + \varepsilon$	10	< 0.0001	< 0.0001	< 0.0001
$F_a = c(T)t^k + F_0(T) + \varepsilon$	17	0.47	0.46	0.00010
$F_a = ct^{k(T)} + F_0(T) + \varepsilon$	17	0.20	0.20	< 0.0001
$F_a = c(T)t^{k(T)} + F_0(T) + \varepsilon$	24	0.0011	0.00042	< 0.0001
$F_a = ce^{kt} + \varepsilon$	2	< 0.0001	< 0.0001	< 0.0001

$F_a = c(T)e^{kt} + \varepsilon$	9	< 0.0001	< 0.0001	< 0.0001
$F_a = ce^{k(T)t} + \varepsilon$	9	Did not converge		
$F_a = c(T)e^{k(T)t} + \varepsilon$	16	< 0.0001	< 0.0001	< 0.0001
$F_a = ce^{kt} + F_0 + \varepsilon$	3	Did not converge		
$F_a = c(T)e^{kt} + F_0 + \varepsilon$	10	< 0.0001	< 0.0001	< 0.0001
$F_a = ce^{k(T)t} + F_0 + \varepsilon$	10	Did not converge		
$F_a = c(T)e^{k(T)t} + F_0 + \varepsilon$	17	< 0.0001	< 0.0001	< 0.0001
$F_a = ce^{kt} + F_0(T) + \varepsilon$	10	Did not converge		
$F_a = c(T)e^{kt} + F_0(T) + \varepsilon$	17	Did not converge		
$F_a = ce^{k(T)t} + F_0(T) + \varepsilon$	17	Did not converge		
$F_a = c(T)e^{k(T)t} + F_0(T) + \varepsilon$	24	Did not converge		

Table S2. Latin square experiment designs for adhesion experiments. Each row of the table corresponds to series of experiments on a sample at a fixed temperature. 7 different contact times were tested at each temperature, denoted by numbers 0 to 6 in the table. 0 corresponds to the shortest contact time (5 s), while 6 corresponds to the longest contact time (625 s). For each temperature a different row was picked from the table. On each row, each contact time was repeated 6 times, giving a total of 7 × 6 = 42 measurements per sample/temperature. Each row has the property that the first-order carryover effects are balanced e.g. experiment 0 is followed by each one of the experiments 1 – 6, except the last experiment of the row that would be followed by the first experiment of the row. The table was constructed by generating six 7-by-7 square matrices with an increasing $n = 1 \ldots 6$, where each cell on row i and column j has the value of $i \times n + j - 2 \bmod 7$. The matrices are then placed side by side. The construction works as long as the number of different contact times is a prime number.

$n = 1$							$n = 2$							$n = 3$							$n = 4$							$n = 5$							$n = 6$						
0	1	2	3	4	5	6	1	3	5	0	2	4	6	2	5	1	4	0	3	6	3	0	4	1	5	2	6	4	2	0	5	3	1	6	5	4	3	2	1	0	6
1	2	3	4	5	6	0	2	4	6	1	3	5	0	3	6	2	5	1	4	0	4	1	5	2	6	3	0	5	3	1	6	4	2	0	6	5	4	3	2	1	0
2	3	4	5	6	0	1	3	5	0	2	4	6	1	4	0	3	6	2	5	1	5	2	6	3	0	4	1	6	4	2	0	5	3	1	0	6	5	4	3	2	1
3	4	5	6	0	1	2	4	6	1	3	5	0	2	5	1	4	0	3	6	2	6	3	0	4	1	5	2	0	5	3	1	6	4	2	1	0	6	5	4	3	2
4	5	6	0	1	2	3	5	0	2	4	6	1	3	6	2	5	1	4	0	3	0	4	1	5	2	6	3	1	6	4	2	0	5	3	2	1	0	6	5	4	3
5	6	0	1	2	3	4	6	1	3	5	0	2	4	0	3	6	2	5	1	4	1	5	2	6	3	0	4	2	0	5	3	1	6	4	3	2	1	0	6	5	4
6	0	1	2	3	4	5	0	2	4	6	1	3	5	1	4	0	3	6	2	5	2	6	3	0	4	1	5	3	1	6	4	2	0	5	4	3	2	1	0	6	5
